# Origin and Formation Mechanism Investigation of Compound Precipitation from the Traditional Chinese Prescription Huang-Lian-Jie-Du-Tang by Isothermal Titration Calorimetry

**DOI:** 10.3390/molecules22091456

**Published:** 2017-09-01

**Authors:** Hui Wang, Tong Li, Hongjun Xiang, Xinyu Zhang, Kang Fang, Gaorong Wu, Mengmeng Yan, Nannan Xue, Meng Chen, Tianxin Xie, Yuzhong Zhang, Penglong Wang, Haimin Lei

**Affiliations:** 1 School of Chinese Pharmacy, Beijing University of Chinese Medicine, Beijing 100102, China; 15652387323@163.com (H.W.); lt1755258545@163.com (T.L.); iloveygr@163.com (H.X.); xinyums@126.com (X.Z.); 18361466994@163.com (K.F.); gaorongwu@163.com (G.W.); yanmengmeng@bucm.edu.cn (M.Y.); 18810612758@163.com (N.X.); 18702267252@163.com (M.C.); xietx1993@163.com (T.X.); 2 Department of Pathology, Beijing University of Chinese Medicine, Beijing 100102, China; zyz100102@126.com

**Keywords:** compounds in the form of precipitation (CFP), Huang-Lian-Jie-Du-Tang (HLJDT), separated prescriptions, isothermal titration calorimetry (ITC), neuroprotective effect

## Abstract

Previous studies have shown that compounds in the form of precipitate (CFP) from Huang-Lian-Jie-Du-Tang (HLJDT) were stable, and the CFP content reached 2.63% of the whole decoction and had good neuroprotective effects. However, there has been no research on their specific source. In this study, it was found that HLJDT CFP mainly came from the reaction of *Scutellaria baicalensis* and *Coptis chinensis* by studying the separated prescription components (accounting for 81.33% of HLJDT CFP). Unlike previous studies on HLJDT CFP, in this research the chemical composition of *Scutellaria baicalensis*–*Coptis chinensis* (SB–CC) CFP was identified by high performance liquid chromatography coupled with mass spectrometry (HPLC-MS^n^), which further proved that the main source of HLJDT CFP was *Scutellaria baicalensis*–*Coptis chinensis* CFP compared with previous HLJDT CFP studies. To explain the reaction mechanism between the decoctions of *Scutellaria baicalensis* and *Coptis chinensis*, isothermal titration calorimetry (ITC) was used to analyze their binding heat and the thermodynamic parameters (Δ*H*, Δ*S*, Δ*G*, n, Ka) of the reaction between baicalin and berberine, which are the main components of *Scutellaria baicalensis* and *Coptis chinensis*, respectively. The results showed that the reaction between decoctions of *Scutellaria baicalensis* and *Coptis chinensis* was exothermic and the reaction between baicalin and berberine was a spontaneous and enthalpy-driven chemical reaction, the binding ratio being 1:1. In addition, HLJDT CFP (EC_50_ = 14.71 ± 0.91 µg/mL) and SB-CC CFP (EC_50_ = 6.11 ± 0.12 µg/mL) showed similar protective activities on PC12 cells injured by cobalt chloride (CoCl_2_). This study provided a new angle to research on the main chemical components and therapeutic values of CFP in Traditional Chinese Medicine compounds.

## 1. Introduction

The existence of compounds in the form of precipitate (CFP) is widespread in the decoctions of Traditional Chinese Medicines, such as Huang-Lian-Jie-Du-Tang [1], Ge-Gen-Qin-Lian-Tang [2], etc. Through the long-term clinical application, some researchers have noticed that it is not easy to avoid the production of sediment in the decoction process [3]. CFP are always abandoned along with the dregs of a decoction in clinical application, which may reduce the amount of active compounds in drugs and lower their efficacy [4]. Recently, studies on the chemical compositions and pharmacological activities of CFP have been carried out by some academics, and they have found that these precipitates contain a lot of bioactive compounds [5,6]. Therefore, it is absolutely essential to conduct a systematic study on CFP in terms of its sources, pharmacological activities and reaction mechanisms.

Huang-Lian-Jie-Du-Tang (HLJDT) is a Traditional Chinese Medicine prescription which is widely used in the clinic. It is composed of *Coptis chinensis*, *Phellodendron chinense*, *Scutellaria baicalensis* and *Gardenia jasminoides* in a proportion of 3:2:2:3. It was widely used in the past for purging heat by removing toxins [7] and now widely used for protecting neurons, treating arthritis, tumors, cardiovascular diseases and so on [8,9,10,11,12]. HLJDT produces a large amount of precipitate during the process of decocting. However, the truth was most studies focused only on the supernatant and the changes of its chemical components and pharmacological activities [13,14]. In previous studies, we concentrated on the formation rate, compositions, and neuroprotective effects of HLJDT CFP, the results of which proved that the HLJDT CFP was both stable and controllable. The formation rate of HLJDT CFP even reached 2.63 ± 0.21% during the process of decoction [15]. Compared with the supernatant, the CFP showed better neuroprotective effects on PC12 cells injured by CoCl_2_. Based on the facts mentioned above, a further study to find out the specific sources of HLJDT CFP was carried out.

In this study, the source of HLJDT CFP was identified according to the separated prescription components. We first selected and mixed every two herbal medicines in a non-woven bag (pore diameter < 30 µm) respectively, and then decocted the mixtures. Later on, the compositions of SB-CC CFP were identified by HPLC-MS^n^ and proved to be almost the same as that of HLJDT CFP [15], thus confirming SB-CC CFP as the main source of HLJDT CFP.

Isothermal titration calorimetry (ITC) was used in order to explore the mechanism of formation of the CFP [16,17,18,19,20,21,22,23,24]. ITC is a physical technique used to determine the thermodynamic parameters of interactions in solution. Based on binding heat, thermodynamic parameters such as enthalpy change (Δ*H*), entropy change (Δ*S*), Gibbs free energy change (Δ*G*), the estimate of stoichiometric ratio (n) and the binding constant (Ka) were calculated using the Nano Analyze software. Through analyzing these parameters, the thermodynamic principles of binding could be elucidated. If Δ*G* is below zero, the reaction is spontaneous and the higher the absolute value of Δ*G*, the easier it is for a reaction to happen. The estimated stoichiometric ratio (n) reflects the binding ratio of two substances as a larger Ka indicates a stronger binding force between two substances [20]. By analysing the relationship between Δ*H* and *T*Δ*S*, the driving form of precipitation could be identified. A negative enthalpy and positive entropy suggest an advantageous contribution to reactions [16]. A huge energy change was observed in the reaction process between the decoctions of *Scutellaria baicalensis* and *Coptis chinensis* and the changes found were very large, indicating the existence of chemical reactions between the main components of *Scutellaria baicalensis* and *Coptis chinensis*. As the HPLC-MS^n^ result showed that the main compositions of SB-CC CFP were baicalin and berberine, respectively, the titration between baicalin and berberine was determined by ITC. This study probed into the reaction type of single components from the perspective of molecular thermodynamics.

PC12 cells injured by CoCl_2_ is frequently used as a model for screening new agents for the intervention on ischemic brain injury [25]. In this paper, the protective effects of CFP against neurotoxicity were evaluated by a cholecystokinin-octopeptide (CCK-8) assay on differentiated PC12 cells [26,27,28,29].

## 2. Results

### 2.1. The Source of HLJDT CFP 

**Figure 1:**
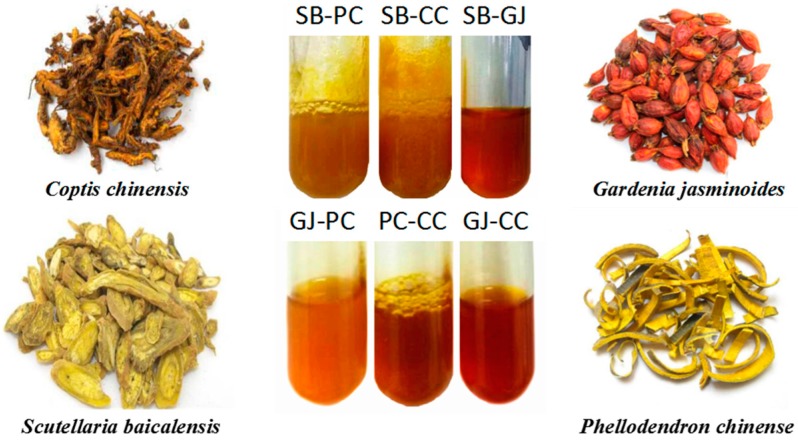
The experimental phenomena of SB-PC SB-CC, SB-GJ, GJ-PC, PC-CC, GJ-CC.

The source of HLJDT CFP was preliminarily determined by studying the separated prescription ingredients. The results were divided into seven groups: HLJDT, *Scutellaria baicalensis-Coptis chinensis* (SB-CC), *Scutellaria baicalensis-Phellodendron chinense* (SB-PC), *Scutellaria baicalensis-Gardenia jasminoides* (SB-GJ), *Gardenia jasminoides-Coptis chinensis* (GJ-CC), *Gardenia jasminoides-Phellodendron chinense* (GJ-PC) and *Phellodendron chinense-Coptis chinensis* (PC-CC). As shown in Table 1, apart from HLJDT CFP, SB-CC and SB-PC, the other groups produced almost no precipitation. SB-CC CFP content accounted for 81.33% in HLJDT CFP. In other words, the HLJDT CFP mainly came from the binding of *Scutellaria baicalensis* and *Coptis chinensis*. In addition, the experimental phenomena were shown in Figure 1. There was a lot of precipitation in the decoction of *Scutellaria baicalensis* and *Coptis chinensis*. The other groups produced less or no precipitation.

### 2.2. HPLC-MS^n^ Analysis of the Constituents from HLJDT CFP and SB-CC CFP

**Figure 2:**
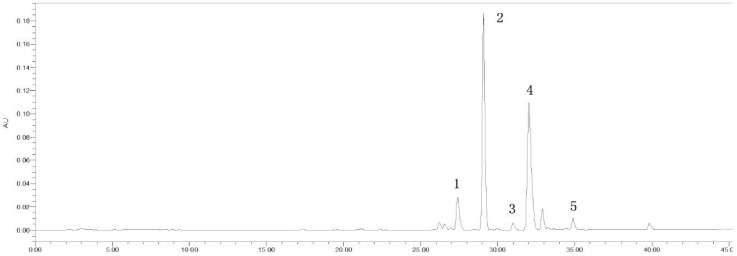
The HPLC chromatogram of SB-CC CFP. The numbers 1 to 5 represent five major chemical constituents.

HPLC-MS^n^ was used to identify the main constituents of SB-CC CFP (Figure 2). As shown in Table 2, five constituents were characterized and determined based on their retention behaviors and MS data. The result showed that the main ingredients of SB-CC CFP were baicalin (2) and berberine (4), which was consistent with the results of previous studies on the chemical substances of HLJDT CFP [15], proving the interactions between Scutellaria baicalensis and Coptis chinensis to be the main source of HLJDT CFP.

### 2.3. Formation Mechanism Test Based on ITC

#### 2.3.1. Titration of Decotions of *Scutellaria baicalensis* and *Coptis chinensis*

**Figure 3:**
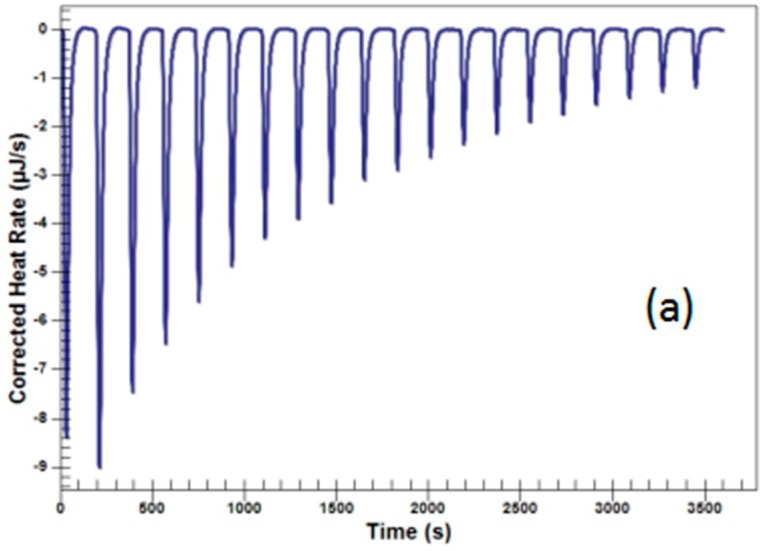

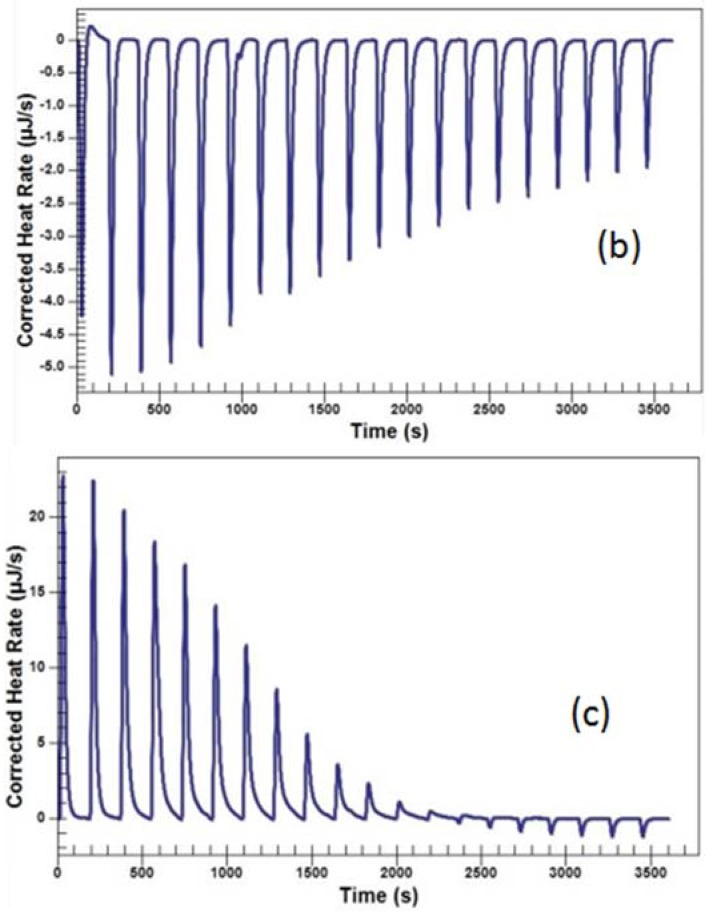
The reactive profiles of colliquefaction (**a**) Calorimetric titration of deionized water with *Scutellaria baicalensis* decoction; (**b**) Calorimetric titration of deionized water with *Coptis chinensis* decoction; (**c**) Calorimetric titration of *Scutellaria baicalensis* decoction with *Coptis chinensis* decoction.

This part consisted of three experiments: the titrations of *Coptis chinensis* decoction into *Scutellaria baicalensis* decoction, *Coptis chinensis* decoction into deionized water and *Scutellaria baicalensis* decoction into deionized water. The latter two titrations were as blank controls. Their binding heats were measured directly by ITC and the data are summarized in Figure 3. The left panel (Figure 3a) shows the corrected heat rates of the direct titration of *Scutellaria baicalensis* into deionized water. The middle panel (Figure 3b) shows the corrected heat rates of the direct titration of *Coptis chinensis* decoction into deionized water. These two were a dilution process and were endothermic. The right panel (Figure 3c) shows the titration of *Coptis chinensis* decoction into *Scutellaria baicalensis* decoction, which was an exothermic process. From these three experiments, it could be seen that the heat quantity in the titrations of *Coptis chinensis* decoction into *Scutellaria baicalensis* decoction was much larger than that of *Scutellaria baicalensis* or *Coptis chinensis* decoction into deionized water, indicating of the existence of interactions between the components of *Coptis chinensis* and *Scutellaria baicalensis* when they were blended.

Moreover, it was highly likely to be a chemical reaction rather than a physical one. All the heat quantities gathered in this study were summarized in Table 3.

#### 2.3.2. Titration of the Solutions of Baicalin and Berberine

**Figure 4:**
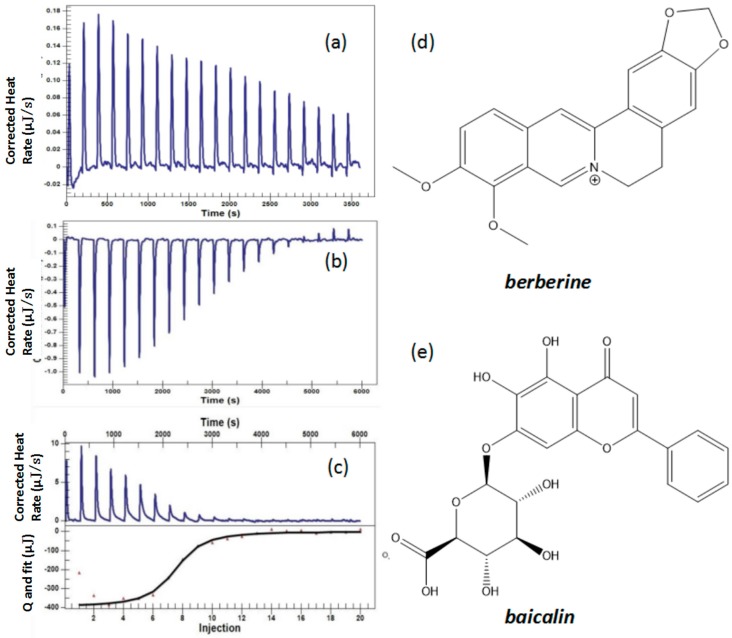
The reactive profiles of colliquefaction (**a**) Calorimetric titration of deionized water with 0.04 mmol/L baicalin solution; (**b**) Calorimetric titration of deionized water with 0.4 mmol/L berberine solution; (**c**) Clorimetric titration of 0.04 mmol/L baicalin solution with 0.4 mmol/L berberine solution and the heating curve of baicalin solution titrated by berberine solution; (**d**) Structure of berberine; (**e**) Structure of baicalin.

This part also consisted of three experiments: the titration of baicalin solution (0.04 mmol/L) into deionized water, berberine solution (0.4 mmol/L) into deionized water and berberine solution (0.4 mmol/L) into baicalin solution (0.04 mmol/L). The binding heats were measured directly by ITC. The binding heat of berberine titrated into deionized water was set as the benchmark, and the Nano Analyze software was used to fit the heating curve of the titration processes, all of which were summarized in Figure 4. It was shown that the direct titration of baicalin solution into deionized water was a very weak exothermic process (Figure 4a); the direct titration of berberine solution into deionized water, just like *Coptis chinensis*, was also an endothermic dilution process (Figure 4b). Figure 4c shows the titration of berberine solution into baicalin solution, which gave off a lot of heat. The heat quantity of berberine titrating baicalin was larger than that of the titration of berberine or baicalin into water (Table 3). The following curve shows the binding thermodynamic parameters (Δ*H*, Δ*S*, Δ*G*, n, Ka, Kd) of baicalin and berberine, summarized in Table 4. Ka = 1.228 × 10^6^ 1/M, Δ*G* = −35.573 kJ/mol, indicating the spontaneity of the reaction between baicalin and berberine. However, as the absolute value of Δ*G* was not very high, this reaction was not very violent. As shown in Table 4, the estimated stoichiometric ratio (n) was 1.002, indicating the binding ratio of baicalin and berberine was 1:1, which conformed to the result of baicalin-berberine complex ^1^H-NMR and ^13^C-NMR test [15]. Δ*H* = −371.6 kJ/mol, −TΔ*S* = 336.027 kJ/mol, which indicated that most of the binding energy was enthalpy, as the entropy change (Δ*S*) of binding contributed unfavorably [16,24]. In other words, this was a chemical action, like the formation of hydrogen bonds, or dipole dipole interactions [30], rather than a physical combination driven by hydraulic force [31]. The chemical structures of baicalin and berberine are shown in Figure 4d,e, respectively.

### 2.4. Protective Effect of the HLJDT CFP and SB-CC CFP on PC12 Cells Injured by CoCl_2_

To evaluate the neuroprotective effects of the HLJDT and the SB-CC, the four samples were tested on neuronal-like PC12 cells induced by CoCl_2_. The results (Table 5) showed that all of them presented protective effects on injured PC12 cells. The results of the neuroprotective effects were ranked as follows: SB-CC CFP > HLJDT CFP > HLJDT supernatant > SB-CC supernatant. The similar effects of HLJDT CFP and SB-CC CFP also indicated the existence of similar actives.

**Figure 5:**
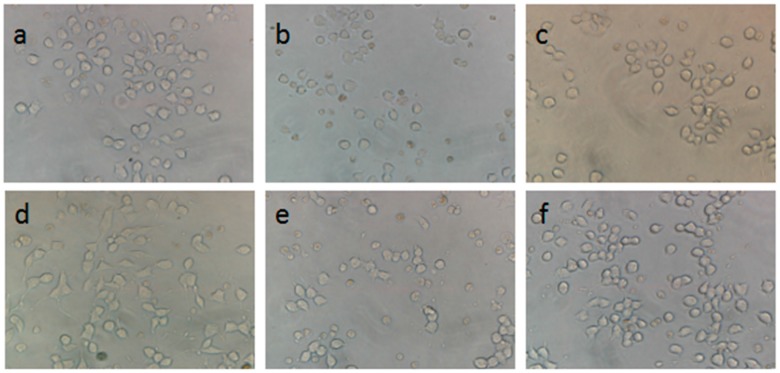
Morphological changes of PC12 cells under a light microscope. (**a**) Control group; (**b**) Model group; (**c**) Pretreatment with 30 μg/mL of HLJDT supernatant then injured by CoCl_2_; (**d**) Pretreatment with 30 μg/mL of HLJDT CFP then injured by CoCl_2_; (**e**) Pretreatment with 30 μg/mL of SB-CC supernatant then injured by CoCl_2_ and (**f**) Pretreatment with 30 μg/mL of SB-CC CFP then injured by CoCl_2_ (×400).

Under a light microscope, the normal differentiated PC12 cells were shown to display complete and distinct edges (Figure 5a). In contrast, the number of cells with 200 mM CoCl_2_ for 12 h was reduced and dendritic networks disappeared (Figure 5b). Cells pretreated with 30 μg/mL HLJDT supernatant and SB-CC supernatant showed weak effects compared to model cells (Figure 5c,e). Cells pretreated with 30 μg/mL of HLJDT CFP and SB-CC CFP evidently showed improvement of the morphological manifestations of the cells and the number of neurite-bearing cells compared to model cells (Figure 5d,f).

## 3. Discussion

The methods of separated prescription mixing and HPLC-MS^n^ were used to identify the source of the CFP. Then, the reaction mechanism of the CFP was revealed by ITC. The model of neuronal-like PC12 cells injured by CoCl_2_ was chosen to evaluate the neuroprotective activity of the CFP. In order to obtain the CFP, we first selected and mixed every two herbal medicines in a non-woven bag, respectively, and then decocted and centrifuged the decoctions after cooling to room temperature. The approximate formation rates of the seven batches of CFP proved that HLJDT CFP (the formation rate = 2.49 ± 0.12%) mainly came from the SB-CC CFP (the formation rate = 4.07 ± 0.20%).

HPLC-MS^n^ was used to identify the substances in CFP. The main components of SB-CC CFP were baicalin and berberine, which conformed to the results of previous research [15]. The results indicated that the main chemical substances of SB-CC CFP and HLJDT CFP were similar. This validated the viewpoint that HLJDT CFP mainly came from SB-CC CFP.

Isothermal titration calorimetry (ITC) was used to evaluate reaction mechanism between *Scutellaria baicalensis* and *Coptis chinensis*. In a reaction, if Δ*H* < 0, −*T*Δ*S* < 0, then the binding energy is favored by enthalpic and entropic contributions; If Δ*H* < 0, −*T*Δ*S* > 0, there will be two cases. The first is |Δ*H*| > *T*|Δ*S*|, the reaction can happen spontaneously and it is favored by enthalpic contribution. Contrarily, the second is |Δ*H*| < *T*|Δ*S*|, and the reaction will not happen; If Δ*H* > 0, −*T*Δ*S* > 0, the reaction will also not happen; If Δ*H* > 0, −*T*Δ*S* < 0, and at the same time |Δ*H*| < *T*|Δ*S*|, the reaction could happen spontaneously and this reaction is favored by entropic contribution [17,18].

The results showed that during the titration process of *Scutellaria baicalensis* and *Coptis chinensis*, the energy level changed significantly, indicating chemical reactions between the main components of *Scutellaria baicalensis* and *Coptis chinensis*. During the titration process of baicalin and berberine, |Δ*H*| > *T*|Δ*S*|, Δ*S <* 0, indicating that the reaction was enthalpy-driven, and the entropy change (Δ*S*) of binding provided an unfavorable contribution. Chemical reactions happened and some internal component(s) changed. The results confirmed that the reaction between baicalin and berberine was a complexing reaction [15]. The results of the nerve activity tests showed that all of HLJDT CFP, HLJDT supernatant, SB-CC CFP and SB-CC supernatant presented protective effects on differentiated PC12 cells injured by CoCl_2_. The CFP had better effects than supernatant, proving that CFP contained more amount of the effective components.

## 4. Materials and Methods

### 4.1. Source of HLJDT CFP

Four different Traditional Chinese Medicines were purchased from Beijing Tong Ren Tang Group Co. (Beijing, China), a Chinese pharmaceutical company, and the four kinds of medicinal herbs were identified, *Scutellaria baicalensis* was the dried root of *Scutellaria baicalensis* Georgi; *Coptis chinensis* was the dried rhizome of *Coptis chinensis* Franch; *Phellodendron chinense* was the dried bark of *Phellodendron chinense* Schneid; *Gardenia jasminoides* was the dried ripe fruits of *Gardenia jasminoides* Ellis. We selected and arranged each combination of two herbal medicines from the above four in non-woven bags (pore diameter < 30 µm) respectively, and then decocted each one with eight times the amount of water for 30 min. We thus obtained gained seven groups: HLJDT, *Scutellaria baicalensis-Coptis chinensis* (SB-CC), *Scutellaria baicalensis-Phellodendron chinense* (SB-PC), *Scutellaria baicalensis-Gardenia jasminoides* (SB-GJ), *Gardenia jasminoides-Coptis chinensis* (GJ-CC), *Gardenia jasminoides-Phellodendron chinense* (GJ-PC) and *Phellodendron chinense-Coptis chinensis* (PC-CC) by centrifugation at 4000 r/min for 15 min after cooling to room temperature. The supernatant was concentrated and the CFP were dried at 30 °C. The precipitation rate (%) was calculated as follows:

Precipitation rate% = (separated precipitation CFP weight/herbal weight) × 100%.(1)

The proportion of separated precipitation CFP among the HLJDT CFP (%) was calculated by the following equation:

Separated precipitation CFP among HLJDT CFP % = (separated prescription CFP weight/HLJDT CFP weight) × 100%.(2)

### 4.2. HPLC-MS^n^ Analysis of the Constituents of HLJDT CFP and Scutellaria baicalensis–Coptis chinensis CFP

The HPLC-MS^n^ analysis was performed with an Agilent 1100 LC system equipped with an LC/MSD Trap XCT Plus mass spectrometer (Agilent Technologies, Santa Clara, CA, USA). A TC-C18 (4.6 mm × 250 mm, 5 μm) column (Agilent) was used for analysis. The column temperature was kept at 30 °C. The mobile phase consisted of acetonitrile (A) and water containing 0.2% phosphoric acid (B). The following gradient condition was used: 0–10 min, 5–15% A; 10–30 min, 15–30% A; 30–42 min, 30–42% A; 42–45 min, 42–48% A; 45–50 min, 48–60% A; and 50–60 min, 60–90% A. The mobile phase flow rate was 1 mL/min and the sample injection volume was 10 μL. The detected wavelength was 238 nm. Mass spectra were acquired in positive and ion modes with an ESI source in the range of *m*/*z* 100 to 1000. The ESI-MS conditions were that the nebulizer pressure at 45 psi and nitrogen as the drying gas at a flow rate of 10 L/min with a temperature of 350 °C. The capillary voltage was set at 3500 V. Data were acquired by use of the Agilent Chemstation software (Agilent Technologies).

### 4.3. Formation Mechanism Test Based on ITC

All ITC experiments were performed with an Auto-ITC isothermal titration calorimeter (TA Instruments, Shanghai, China). The extraction solutions of *Scutellaria baicalensis* and *Coptis chinensis* were respectively packaged in a non-woven bag (pore diameter < 30 µm) and decocted with eight times the amount of water for 30 min. The extract was diluted to a suitable concentration by adding deionized water. The baicalin was dissolved to 0.04 mmol/L with deionized water and berberine was dissolved to 0.4 mmol/L. All ITC experiments were performed at 303.00 K. To carry out the titration, the injector was filled with extract of *Coptis chinensis* test solution and the working cell with extract of *Scutellaria baicalensis* test solution. In the monomer composition experiments, the injector was filled with berberine test solution and the working cell with baicalin test solution. The ITC titrations were carried out at the condition of 250 r/min. The titration was sustained for 20 injections with injections of 2.5 μL, and an interval of 180 s between injections to ensure complete equilibration. A background titration, performed with identical extract of *Coptis chinensis* or berberine in sampling needle but with the sample cell filled just with the deionized water, was subtracted from the main experiment to account for the heat of dilution.

After the titration, the Nano Analyze software was used to analyze the data. It automatically searched and fitted the curve of titrations and calculated thermodynamic parameters after we input sample concentrations. The thermodynamic parameters consists of Δ*H*, ΔS, n, Ka; the Gibbs free energy change (Δ*G*) were calculated using the standard thermodynamic equation: Δ*G* = − RTlnK = Δ*H* − *T*Δ*S*.

At present, ITC is mainly used in molecular interaction, characterization and enzyme dynamics studies, especially used to characterize the binding between a drug molecule and a receptor protein, where almost all combinations are exothermic. From an energetic standpoint, the binding of molecules or molecules to receptors would lead to a decrease in energy, which was the driving force and trend of binding. In order to reduce the energy of the system, the binding would generally be exothermic, otherwise it would not combine.

### 4.4. Protective Effect of the HLJDT CFP and Scutellaria baicalensis–Coptis chinensis CFP on Injured PC12 Cells

PC12 cells were cultivated in Rosewell Park Memorial Institute (RPMI) 1640 medium supplemented with 5% (*v*/*v*) fetal bovine serum, 10% (*v*/*v*) horse serum and 100 U/mL penicillin-streptomycin (Thermo Technologies, New York, NY, USA) at 37 °C in a humidified atmosphere of 5% CO_2_. When the coverage area of cells >80%, the original medium was removed and cells were cultured with the serum-free medium for 14 h. Then the cells were suspended in 1640 medium supplemented with 10% (*v*/*v*) fetal bovine serum, and seeded into poly-l-lysine-coated 96-well culture plates at 7 × 10^3^ cells/well, differentiate and treated with 50 n g/mL nerve growth factor (NGF) for 48 h. After these, the differentiated PC12 cells were pretreated with various concentrations (60, 30, 15, 7.5, 3.75 µg/mL) of samples for 36 h. All measurements were performed after the cells were injured by CoCl_2_ (final concentration, 200 mM) for 12 h except control differentiated cells. After adding CCK-8 solution (10 µL, 5 mg/mL) to each well, the plate was incubated for a further 4 h at 37 °C in a humidified atmosphere of 5% CO_2_. Then the absorbance at 490 nm was measured with a BIORAD 550 spectrophotometer (Bio-Rad, Berkeley, CA, USA). The proliferation rates of damaged PC12 cells were calculated by the formula [OD_490_ (Sample) − OD_490_ (CoCl_2_)]/[OD_490_ (NGF) − OD_490_ (CoCl_2_)] × 100%.

## 5. Conclusions

In conclusion, the source of HLJDT CFP, which is the result of the reaction between *Scutellaria baicalensis* and *Coptis chinensis*, was identified by studying the separated prescriptions and HPLC-MS^n^. In order to find out formation mechanism of HLJDT CFP, ITC was used. The results indicated that there were some reactions between the main components of *Scutellaria baicalensis* and *Coptis chinensis*. The combination of baicalin and berberine was very powerful and the reaction between them was an enthalpy-controlled chemical reaction. All samples showed protective effects against cobalt chloride-injured neurotoxicity in differentiated PC12 cells. In addition, SB-CC CFP (EC_50_ = 6.11 ± 0.12 µg/mL) displayed the best neuroprotective activity.

## Figures and Tables

**Table 1:** The formation rate of HLJDT CFP and seven separated prescriptions from different batches.

No.	Batch	*Coptis chinensis*	*Scutellaria baicalensis*	*Phellodendron chinense*	*Gardenia jasminoides*	Total Weight	Precipitate Weight	Precipitation Rate	Separated Precipitate CFP among HLJDT CFP
**HLJDT**	1	9.01 g	6.00 g	5.98 g	9.00 g	29.99 g	0.78 g	2.60%	
	2	8.97 g	5.99 g	5.99 g	9.01 g	29.98 g	0.71 g	2.37%	
	3	8.99 g	6.01 g	5.98 g	9.02 g	30.00 g	0.75 g	2.50%	
	average					29.99 ± 0.01 g	0.75 ± 0.04 g	2.49 ± 0.12%	
**SB-CC**	1	9.00 g	6.01 g			15.01 g	0.62 g	4.13%	
	2	8.99 g	5.99 g			14.98 g	0.58 g	3.87%	
	3	9.01 g	5.98 g			14.99 g	0.63 g	4.20%	
	average					14.99 ± 0.02 g	0.61 ± 0.03 g	4.07 ± 0.20%	81.33%
**SB-PC**	1		5.99 g	5.98 g		11.97 g	0.06 g	0.51%	
	2		6.01 g	6.00 g		12.01 g	0.05 g	0.42%	
	3		6.03 g	5.99 g		12.02 g	0.06 g	0.50%	
	average					12.00 ± 0.03 g	0.06 ± 0.01 g	0.48 ± 0.06%	8.00%
**GJ-CC**	1	9.02 g			9.03 g	18.05 g	— ^a^	—	
	2	9.00 g			9.00 g	18.00 g	—	—	
	3	9.02 g			8.99 g	18.01 g	—	—	
	average					18.02 ± 0.03 g	—	—	
**GJ-PC**	1			6.00 g	8.98 g	14.98 g	—	—	
	2			6.01 g	8.99 g	15.00 g	—	—	
	3			5.98 g	9.00 g	14.98 g	—	—	
	average					14.99 ± 0.01 g	—	—	
**SB-PC**	1		6.03 g		8.99 g	15.02 g	—	—	
	2		5.98 g		9.01 g	14.98 g	—	—	
	3		6.01 g		9.03 g	15.04 g	—	—	
	average					15.01± 0.04 g	—	—	
**PC-CC**	1	8.98 g		6.01 g		14.99 g	—	—	
	2	9.01 g		6.02 g		15.03 g	—	—	
	3	9.00 g		5.97 g		14.97 g	—	—	
	average					15.00± 0.01 g	—	—	

—^a^ represented precipitation weight <0.05 g or Precipitation Rate <0.33%.

**Table 2:** ESI-MS^n^ ions of the identified compounds.

Peak	Tr (min)	Compounds	Ms (*m*/*z*)	Ms^2^ (*m*/*z*)
**1**	27.401	Coptisine	319.81 [M]^+^	291.87 [M − C_2_H_4_]^+^
**2**	29.001	Baicalin	446.97 [M + H]^+^	270.75 [M + H − GLU]^+^
**3**	31.008	Palmatine	351.92 [M]^+^	335.86 [M − CH_4_]^+^
				307.85 [M − CH_4_ − C_2_H_4_]^+^
**4**	32.034	Berberine	335.85 [M]^+^	319.81 [M − CH_4_]^+^
				291.82 [M − CH_4_ − C_2_H_4_]^+^
**5**	34.899	Wogonoside	460.97 [M + H]^+^	284.80 [M + H − Glu]^+^

**Table 3:** Binding heat of all titrations.

No.	*Coptis chinensis* into *Scutellaria baicalensis* (μJ)	*Scutellaria baicalensis* into Water (μJ)	*Coptis chinensis* into Water (μJ)	Berberine into Baicalin (μJ)	Berberine into Water (μJ)	Baicalin into Water (μJ)
1	—	—	—	—	—	—
2	594.11	−228.2	−122.09	313.21	−22.00	−3.35
3	629.88	−189.2	−125.01	358.41	−23.77	−4.36
4	720.57	−161.6	−123.93	325.81	−24.89	−3.85
5	554.46	−139.1	−119.18	323.80	−24.49	−3.55
6	483.12	−119.7	−115.41	313.13	−22.15	−3.50
7	411.30	−104.6	−100.42	219.95	−19.87	−3.20
8	315.20	−94.20	−99.69	126.31	−18.40	−3.13
9	215.62	−86.06	−93.10	61.22	−14.93	−2.81
10	147.73	−74.72	−87.18	44.42	−12.87	−2.58
11	92.88	−69.86	−81.03	27.56	−9.98	−2.86
12	55.09	−64.10	−76.37	17.60	−8.68	−2.60
13	26.51	−57.33	−73.13	0.78	−7.15	−2.34
14	6.84	−51.45	−66.79	−15.98	−4.01	−2.14
15	−6.99	−45.71	−63.61	−4.83	−2.49	−2.00
16	−18.57	−41.89	−62.47	−7.92	0.06	−2.01
17	−25.25	−36.65	−59.78	5.23	−0.80	−2.07
18	−29.76	−33.33	−56.14	0.85	0.36	−1.75
19	−31.58	−30.35	−52.34	3.31	1.80	−1.16
20	−33.05	−27.86	−50.15	−10.56	2.11	−1.70

‘—’ the deviation of the first drops was large, so they were eliminated.

**Table 4:** Binding thermodynamics of baicalin and berberine.

No.	Δ*H* (kJ/mol)	−*T*Δ*S* (kJ/mol)	Δ*G* (kJ/mol)	n	Ka (1/M)	Kd (M)
**Berberine to baicalin**	−371.6	336.027	−35.573	1.002	1.228 × 10^6^	8.143 × 10^−7^

**Table 5:** The protective effect of HLJDT CFP, HLJDT supernatant, SB-CC CFP and SB-CC supernatant on PC12 cells injured by CoCl_2_ (data are expressed as means ± SD from three separate experiments).

Samples	Proliferation Rate (%)					EC_50_ (µg/mL)
	3.75 µg/mL	7.5 µg/mL	15 µg/mL	30 µg/mL	60 µg/mL	
**HLJDT supernatant**	2.36 ± 0.30	11.46 ± 3.67	46.67 ± 6.06	60.28 ± 3.83	45.24 ± 5.01	28.41 ± 2.61
**HLJDT CFP**	39.49 ± 7.06	53.28 ± 5.57	56.46 ± 5.69	83.18 ± 4.69	19.83 ± 4.20	14.71 ± 0.91
**SB-CC supernatant**	11.43 ± 3.26	14.79 ± 4.01	14.19 ± 0.69	24.16 ± 5.95	10.60 ± 2.93	56.47 ± 2.63
**SB-CC CFP**	32.58 ± 4.44	58.04 ± 5.87	66.28 ± 2.19	123.91 ± 9.41	84.64 ± 4.69	6.11 ± 0.12
